# Outcomes of severely injured pregnant trauma patients: a multicenter analysis

**DOI:** 10.1007/s13304-024-01817-3

**Published:** 2024-03-30

**Authors:** Kyrillos G. Awad, Jeffry Nahmias, Negaar Aryan, Alexa N. Lucas, Nicole Fierro, Navpreet K. Dhillon, Eric J. Ley, Jennifer Smith, Sigrid Burruss, Alden Dahan, Arianne Johnson, William Ganske, Walter L. Biffl, Dunya Bayat, Matthew Castelo, Diane Wintz, Kathryn B. Schaffer, Dennis J. Zheng, Areti Tillou, Raul Coimbra, Rahul Tuli, Jarrett E. Santorelli, Brent Emigh, Morgan Schellenberg, Kenji Inaba, Thomas K. Duncan, Graal Diaz, Erika Tay-Lasso, Danielle C. Zezoff, Areg Grigorian

**Affiliations:** 1 Department of Surgery, Desert Regional Medical Center, Palm Springs, CA USA; 2 Division of Trauma, Burns, and Surgical Critical Care, Department of Surgery, University of California, Irvine Medical Center, 333 The City Blvd West, Suite 1600, Orange, CA 92868-3298 USA; 3 Department of Surgery, Cedars-Sinai Medical Center, Los Angeles, CA USA; 4 Division of Trauma and Critical Care, Harbor-UCLA Hospital, Torrance, CA USA; 5 Department of Trauma, Acute Care Surgery, Surgical Critical Care, Loma Linda Medical Center, Loma Linda, CA USA; 6 Cottage Health Research Institute, Santa Barbara Cottage Hospital, Santa Barbara, CA USA; 7 Trauma and Acute Care Surgery, Scripps Memorial Hospital La Jolla, La Jolla, CA USA; 8 Department of Surgery, Sharp Memorial Hospital, San Diego, CA USA; 9 Department of Surgery, UCLA David Geffen School of Medicine, Los Angeles, CA USA; 10 Riverside University Health System Medical Center, Comparative Effectiveness and Clinical Outcomes Research Center – CECORC, Moreno Valley, CA USA; 11 Division of Trauma, Surgical Critical Care, Burns, and Acute Care Surgery, San Diego School of Medicine, University of California, San Diego, CA USA; 12 The Warren Alpert Medical School of Brown University, Providence, RI USA; 13 Division of Acute Care Surgery, LAC+USC Medical Center, University of Southern California, Los Angeles, CA USA; 14 Department of Trauma, Ventura County Medical Center, Ventura, CA USA

**Keywords:** Severe trauma, Pregnant trauma, Fetus, Mortality, Resuscitative hysterotomy, Fetal delivery

## Abstract

Nearly 10% of pregnant women suffer traumatic injury. Clinical outcomes for pregnant trauma patients (PTPs) with severe injuries have not been well studied. We sought to describe outcomes for PTPs presenting with severe injuries, hypothesizing that PTPs with severe injuries will have higher rates of complications and mortality compared to less injured PTPs. A post-hoc analysis of a multi-institutional retrospective study at 12 Level-I/II trauma centers was performed. Patients were stratified into severely injured (injury severity score [ISS] > 15) and not severely injured (ISS < 15) and compared with bivariate analyses. From 950 patients, 32 (3.4%) had severe injuries. Compared to non-severely injured PTPs, severely injured PTPs were of similar maternal age but had younger gestational age (21 vs 26 weeks, *p* = 0.009). Penetrating trauma was more common in the severely injured cohort (15.6% vs 1.4%, *p* < 0.001). The severely injured cohort more often underwent an operation (68.8% vs 3.8%, *p* < 0.001), including a hysterectomy (6.3% vs 0.3%, *p* < 0.001). The severely injured group had higher rates of complications (34.4% vs 0.9%, *p* < 0.001), mortality (15.6% vs 0.1%, *p* < 0.001), a higher rate of fetal delivery (37.5% vs. 6.0%, *p* < 0.001) and resuscitative hysterotomy (9.4% vs. 0%, *p* < 0.001). Only approximately 3% of PTPs were severely injured. However, severely injured PTPs had a nearly 40% rate of fetal delivery as well as increased complications and mortality. This included a resuscitative hysterotomy rate of nearly 10%. Significant vigilance must remain when caring for this population.

**Supplementary Information:**

The online version contains supplementary material available at 10.1007/s13304-024-01817-3.

## Introduction

Trauma impacts up to 1 in 12 pregnancies [1–7] and is the leading non-obstetric cause of death among pregnant patients in the United States, accounting for just over 20% of maternal deaths [8] and 4000 fetal losses annually in the US [2, 6, 9]. During pregnancy, traumatic injury poses a significant and unique challenge, as it not only involves the well-being of the mother but also the developing fetus. Although the relationship between injury severity and outcomes in the general trauma population has been established, its implications for pregnant trauma patients (PTPs) remain less clear [2, 5, 6, 9–13].

Many physiologic changes that occur during pregnancy have direct effects on both the initial patient presentation as well as management in the acute phase after trauma [5, 6, 14–16]. For instance, the physiological hypervolemia characteristic of pregnancy can mask early signs of maternal hemorrhagic shock until substantial blood loss has occurred. Furthermore, gestational age has been found to influence the severity of fetal and uterine injuries after trauma [14, 15].

Given the inconsistencies and gaps in the current literature [1, 4, 11, 12, 17–20] there is a pressing need for a more comprehensive understanding of the relationship between injury severity and maternal–fetal outcomes in PTPs. This study aims to address this by investigating the impact of injury severity on PTP outcomes. We hypothesize that greater injury severity in PTPs is directly associated with a higher rate of complications and mortality. These data may help inform clinical decision-making, facilitate patient counseling, and potentially contribute to the development of targeted management algorithms for this patient population that historically has been excluded from most trauma research.

## Methods

A post hoc analysis of a multi-institutional retrospective study of PTPs ≥ 18 years of age at 12 Level-I/II trauma centers was performed between 2016 and 2021. Approval for this study was granted by the Institutional Review Board of each participating center with a waiver of informed consent. Exclusion criteria included prisoners. The primary outcome was severe trauma, defined by an injury severity score (ISS) ≥ 15. Secondary outcomes included operative interventions, complications, and mortality. We compared PTPs with and without severe trauma.

Additional variables collected include maternal age, gestational age, injury mechanism, vitals on arrival, and injury profile. Exam findings, including vaginal bleeding and abdominal tenderness on admission, were also evaluated. We also collected maternal outcomes, including maternal mortality, urinary tract infection (UTI), ventilator-associated pneumonia (VAP), and sepsis. Rates of pregnancy-related complications, including fetal delivery, placental injury, and premature rupture of membranes (PROM) were also captured. Laboratory studies collected included complete blood count (CBC), hemoglobin and coagulation studies, prothrombin time and international normalized ratio (PT/INR), partial thromboplastin time (PTT), and fibrinogen. Imaging studies were also collected, including computerized tomography (CT), magnetic resonance imaging (MRI), and ultrasonography.

Descriptive statistics were performed for all variables. Pearson’s chi-squared test was used for categorical variables and were reported as percentages. Continuous data were reported as medians with interquartile range and analyzed using a Mann–Whitney *U* test. All *p* values were two-sided, with a critical significance level of < 0.05. All analyses were performed with IBM SPSS Statistics for Windows (Version 29, IBM Corp., Armonk, NY).

## Results

A total of 950 PTPs were identified. Of these, 32 (3.4%) presented with severe trauma, and 918 (95.6%) did not have severe trauma. The most common mechanism of injury was motor vehicle collision (MVC) in both the non-severe (77.7%) and severely injured (46.9%) cohorts. In both the non-severely injured and severely injured cohorts, approximately two-thirds presented after high-speed collisions at estimated speeds of 25 miles per hour or faster (77.9% vs 46.9%, *p* < 0.001). Although observed in both groups, the severely injured cohort more often presented with vaginal bleeding (9.4% vs 1.5%, *p* < 0.001) (Table 1).

**Table 1:** Demographics, injury characteristics, and presentation findings for PTPs stratified by injury severity score

	ISS < 15 (*n* = 918)	ISS ≥ 15 (*n* = 32)	*p* value
Age, years, median	28 (16, 50)	26 (17, 37)	0.957
Gestational age, weeks, median	26 (1, 41)	20.5 (3, 37)	0.009
Blunt mechanism of injury, n (%)	906 (98.7%)	27 (84.4%)	< 0.001
Ground level fall	49 (5.3%)	0 (.0%)	0.180
Fall from height	19 (2.1%)	1 (3.1%)	0.683
Pedestrian versus auto	36 (3.9%)	8 (18.2%)	< 0.001
Motorcycle collision	2 (0.2%)	1 (3.1%)	< 0.004
MVC, *n* (%)	715 (77.9%)	15 (46.9%)	< 0.001
MVC, high speed*	465 (50.7%)	15 (46.9%)	0.674
Assault	79 (8.6%)	3 (9.4%)	0.879
Other	10 (1.1%)	2 (6.3%)	0.010
Penetrating mechanism of injury, *n* (%)	13 (1.4%)	5 (15.6%)	< 0.001
Gunshot	6 (0.7%)	3 (9.4%)	< 0.001
Stab	4 (0.4%)	2 (6.3%)	< 0.001
Other	3 (0.3%)	0 (0.0%)	.746
Suicide attempt	3 (0.3%)	2 (6.3%)	< 0.001
Domestic violence	66 (7.2%)	4 (12.5%)	.258
Injury severity score, median (IQR)	1 (0)	22 (11)	< 0.001
Vital signs on arrival, median			
SBP, median (mmHg)	122	117	0.514
Heart rate, median (beats per minute)	92	104	0.031
Vaginal bleeding, *n* (%)	14 (1.5%)	3 (9.4%)	< 0.001

*MVC* motor vehicle collision, *IQR* interquartile range, *SBP* systolic blood pressure

*High speed defined as > 25 miles per hour

Severely injured PTPs more commonly sustained injuries to major body regions, including higher rates of intracranial hemorrhage (53.1% vs. 3.5%), as well as chest (56.3% vs. 2.7%), abdominal (46.9% vs 6%), and extremity injury (65.6% vs 6.1%) (all *p* < 0.001) (Table 2). Severely injured PTPs had increased rates of operative intervention (68.8% vs. 3.8%, *p* < 0.001), which notably include a resuscitative hysterotomy (9.4% vs 0%, *p* < 0.001) compared to those without severe trauma. Laparotomy rates were also markedly higher in the severely injured group (21.9% vs 0.8%, *p* < 0.001) (Table 3).

**Table 2:** Patterns of maternal injury stratified by injury severity score

	ISS < 15 (*n* = 918)	ISS ≥ 15 (*n* = 32)	*p* value
Head, *n* (%)	29 (3.2%)	15 (46.9%)	< 0.001
Intracranial hemorrhage	32 (3.5%)	17 (53.1%)	< 0.001
Skull fracture	4 (0.4%)	9 (28.1%)	< 0.001
Neck, *n* (%)	7 (0.8%)	6 (18.8%)	< 0.001
Cervical spine fracture	2 (0.2%)	5 (15.6%)	< 0.001
Cervical ligamentous injury	2 (0.2%)	1 (3.1%)	0.004
BCVI	1 (0.1%)	2 (6.3%)	< 0.001
Chest, *n* (%)	25 (2.7%)	18 (56.3%)	< 0.001
Rib fracture	9 (1.0%)	5 (15.6%)	< 0.001
Pneumothorax	5 (0.5%)	13 (40.6%)	< 0.001
Hemothorax	1 (0.1%)	6 (18.8%)	< 0.001
Sternal fracture	6 (0.7%)	2 (6.3%)	< 0.001
Abdomen, *n* (%)	55 (6.0%)	15 (46.9%)	< 0.001
Liver	3 (0.3%)	6 (18.8%)	< 0.001
Spleen	2 (0.2%)	7 (21.9%)	< 0.001
Renal	1 (0.1%)	0 (0.0%)	0.852
Pancreas	4 (0.4%)	0 (0.0%)	0.708
Bladder	3 (0.3%)	1 (3.1%)	0.016
Stomach	3 (0.3%)	0 (0.0%)	0.746
Small bowel	5 (0.5%)	0 (0.0%)	0.676
Colon	5 (0.5%)	0 (0.0%)	0.676
*Uterine contusion*			
Extremity, *n* (%)	56 (6.1%)	21 (65.6%)	< 0.001
Femur	7 (0.8%)	3 (9.4%)	< 0.001
Humerus	2 (0.2%)	1 (3.1%)	0.004
Tibia/fibula	8 (0.9%)	4 (12.5%)	< 0.001
Pelvis, *n* (%)	7 (0.8%)	8 (25%)	< 0.001

*BCVI*  blunt cerebrovascular injury

**Table 3:** Maternal operations stratified by severity of injury

	ISS < 15 (*n* = 918)	ISS ≥ 15 (*n* = 32)	*p* value
Operation, *n* (%)	35 (3.8%)	22 (68.8%)	< 0.001
Tracheostomy	0 (0.0%)	1 (3.1%)	< 0.001
Laparotomy	7 (0.8%)	7 (21.9%)	< 0.001
Thoracotomy	0 (0.0%)	2 (6.3%)	< 0.001
Craniectomy/ectomy	0 (0.0%)	3 (9.4%)	< 0.001
Vascular	1 (0.1%)	3 (9.4%)	< 0.001
Total hysterectomy	3 (0.3%)	2 (6.3%)	< 0.001
Resuscitative hysterotomy	0 (0.0%)	3 (9.4%)	< 0.001

An increased rate of complications was observed in severely injured PTPs, with higher rates of sepsis (6.3% vs. 0.2%), VAP (3.1% vs. 0%), and UTI (6.3% vs. 0.3%) (all *p* < 0.001) (Table 4). Additionally, severely injured PTPs had increased rates of pregnancy-related complications, including unplanned fetal delivery (37.5% vs. 6.0%, *p* < 0.001), PROM (15.6% vs 2.5%, *p* < 0.001), and placental injury (15.6% vs 4.4%, *p* = 0.003). Maternal mortality also occurred more frequently in the severely injured cohort (15.6% vs 0.1%, *p* < 0.001) (Table 4).

**Table 4:** Maternal complications and fetal assessment stratified by maternal injury severity

	ISS < 15 (*n* = 918)	ISS ≥ 15 (*n* = 32)	*p* value
*Maternal complications*, *n* (%)			
Mortality, *n* (%)	1 (0.1%)	5 (15.6%)	< 0.001
Sepsis	2 (0.2%)	2 (6.3%)	< 0.001
Ventilator associated pneumonia	0 (0.0%)	1 (3.1%)	< 0.001
Acute kidney injury	0 (0.0%)	1 (3.1%)	< 0.001
Deep vein thrombosis	2 (0.2%)	0 (0.0%)	0.792
Urinary tract infection	3 (0.3%)	2 (6.3%)	< 0.001
Fetal delivery, *n* (%)	55 (6.0%)	12 (37.5%)	< 0.001
Viable delivery	53 (5.8%)	10 (31.3%)	< 0.001
Fetal heart rate (beats per minute), median (IQR)	143 (14)	130 (155)	0.897
Formal fetal ultrasound done, *n* (%)	549 (59.8%)	17 (53.1%)	.449
Fetal demise	1 (0.1%)	0 (0.0%)	
Angiography performed, *n* (%)	6 (0.7%)	7 (21.9%)	< 0.001
Fetal assessment with continuous tocometry, *n* (%)	473 (51.5%)	5 (15.6)	< 0.001
Abnormal fetal heart tracing, *n* (%)	98 (14.1%)	7 (53.8%)	< 0.001
Premature rupture of membranes, *n* (%)	26 (2.8%)	5 (15.6%)	< 0.001
Fetal contractions upon arrival, *n* (%)	165 (18%)	2 (6.3%)	0.087
Placental injury, *n* (%)	40 (4.4%)	5 (15.6%)	0.003

*IQR*  interquartile ratio

## Discussion

PTPs represent a distinct trauma population with special considerations taught in Advanced Trauma Life Support (ATLS) to help guide clinical management. However, there is a paucity of research on outcomes for severely injured PTPs. This multicenter study found that severely injured PTPs had significantly worse outcomes compared to their non-severely injured counterparts. Specifically, the severely injured PTPs had increased rates of pregnancy-related complications and maternal mortality. Moreover, severely injured PTPs also had an increased rate of resuscitative hysterotomy, highlighting the potential complexity of managing this population.

Increasing injury severity has been clearly demonstrated to correlate with worse outcomes in non-pregnant trauma patients. Palmer et al. found increased hospital length of stay, rates of ICU admission, and death in adult trauma patients with ISS > 15 [18]. This current study similarly found a significant difference between increased injury severity and worse maternal outcomes in PTPs, including maternal mortality, which aligns with previous smaller studies [5, 6, 10, 15, 19–23]. One notable finding in our study was the high rate of resuscitative hysterotomy in severely injured PTPs. Resuscitative hysterotomy is a life-saving intervention performed in cases of maternal hemodynamic instability, aiming to save the life of the mother in extremis by quickly controlling blood loss, as well as potentially save a viable fetus. Maternal survival following this procedure ranges from 34 to 54% [24]. Fetal survival is less precisely defined in the literature, ranging from 0 to 89% [25]. The relatively high possibility of undergoing such a procedure underscores the importance of early identification and aggressive management of severely injured PTPs. Our results also highlight the necessity for trauma centers to be adequately prepared to provide specialized care for PTPs, which may be aided by a multidisciplinary team of trauma surgeons, obstetricians, and neonatologists.

For a PTP in extremis or nearing cardiac arrest, a timely resuscitative hysterotomy might be the sole lifeline for the fetus. Decisive action is crucial, often within minutes of the patient’s arrival. Key factors predicting survival include the fetus’s gestational age, with those ≥ 24 weeks deemed viable [5]. Signs of fetal distress, such as an alarming fetal heart rate, could warrant an immediate delivery. Utilizing point-of-care ultrasound is crucial to gauge fetal heart activity, identify placental positioning, and detect potential free fluid suggestive of maternal intra-abdominal hemorrhage. With placental abruption being life-threatening and prevalent in up to 50% of severely injured PTPs, it must be rapidly diagnosed and acted upon to give a chance for survival to a potentially viable fetus [26]. Concurrently, it is paramount to have neonatology or pediatric specialists on standby, as delivery is just the beginning of the specialized care required following delivery for situations where the fetus and the mother are in extremis.

The mechanisms of traumatic injury in PTPs may have shifted in recent years. Historically, domestic violence and homicide were considered the leading causes of traumatic maternal mortality [4, 11, 12, 27]. While we did find significantly higher rates of penetrating trauma in the severely injured cohort (15.6% vs. 1.4%, *p* < 0.001), MVCs accounted for nearly half of all severely injured PTPs in the present study. This aligns with most recently published series identifying MVCs as the most common mechanism of injury among PTPs, although these have not specifically focused on severely injured PTPs [2, 3, 5, 13, 15, 19, 22, 28]. Continued efforts of injury prevention and risk mitigation with seatbelt use should, therefore, remain a focus during pregnancy counseling by all healthcare providers. Increased efforts to improve public awareness about the risks of trauma during pregnancy and the use of appropriate safety measures in various settings (e.g., road safety and domestic violence prevention) also appear warranted.

The severity of trauma may impact fetal delivery rates in PTPs. Our prior series focusing on rates of fetal delivery for a broader pregnant cohort found a delivery rate of approximately 10% among viable-aged fetuses [29]. The present study found that PTPs presenting with severe injuries had a fetal delivery rate fivefold higher than non-severely injured PTPs. The increased need for delivery is reflective of both the injury burden of the mother as well as compromised uteroplacental blood flow in patients presenting in extremis, again highlighting the need for timely intervention and a multidisciplinary, team-based approach in the management of these patients.

This study is subject to limitations that merit consideration. First, the retrospective design is subject to inherent limitations. In addition, using multiple institutions introduces variability in data collection and reporting practices across different trauma centers, which could lead to misclassification or other data entry errors. Furthermore, while we used ISS as a surrogate for the severity of trauma, this measure may not fully capture the complexity and variability of injury mechanisms in PTPs. Moreover, the cutoff point of ISS of 15, while commonly used, is not ubiquitous, as some refer to an ISS > 25 as severe trauma [18, 30]. Finally, this study did not account for potential differences in patient characteristics, such as pre-existing health conditions and social determinants of health, which all can influence outcomes [2, 6, 7, 9].

## Conclusion

This multicenter study spanning 5 years of data demonstrated that severely injured PTPs experienced higher rates of complications, fetal delivery, resuscitative hysterotomy, and mortality compared to non-severely injured PTPs. These findings elucidate an opportunity for increased research, guideline development, and opportunities where interdisciplinary care, prevention strategies, and quality improvement initiatives may help reduce trauma in this vulnerable population and mitigate worse outcomes seen in severely injured PTPs.

## Supplementary Information

Below is the link to the electronic supplementary material.

Supplementary file1 (DOCX 14 KB)
