# Supplementary material for: Outcomes of severely injured pregnant trauma patients: a multicenter analysis
Source: Updates Surg. 2024 Mar 30;76(6):2441–7. doi: 10.1007/s13304-024-01817-3 (PMC11541353; doi:10.1007/s13304-024-01817-3)
Supplement: Supplementary file 1 — Supplementary file1 (DOCX 14 KB) [file 13304_2024_1817_MOESM1_ESM.docx]

**Outcomes of Severely Injured Pregnant Trauma Patients: A Multicenter Analysis**

Authors: Kyrillos G Awad DO^a^ : literature search, study design, data collection, data analysis, data interpretation, writing, critical revision

Jeffry Nahmias MD MPHE^b^, : study design, data collection, data analysis, data interpretation, critical revision

Negaar Aryan MD^b^, : study design, data collection, data analysis, data interpretation, critical revision

Alexa N. Lucas MD MBA^b^ , : data collection, data analysis, data interpretation, critical revision

Nicole Fierro MD^c^, : data collection, data analysis, data interpretation, critical revision

Navpreet K. Dhillon MD^c^, : data collection, data analysis, data interpretation, critical revision

Eric J. Ley MD^c^, : data collection, data analysis, data interpretation, critical revision

Jennifer Smith MD^d^, : data collection, data analysis, data interpretation, critical revision

Sigrid Burruss MD^d^ , : data collection, data analysis, data interpretation, critical revision

Alden Dahan BS^e^, : data collection, data analysis, data interpretation, critical revision

Arianne Johnson PhD^f^, : data collection, data analysis, data interpretation, critical revision

William Ganske MD^f^, : data collection, data analysis, data interpretation, critical revision

Walter L. Biffl MD^g^, : data collection, data analysis, data interpretation, critical revision

Dunya Bayat MPH^g^, : data collection, data analysis, data interpretation, critical revision

Matthew Castelo^g^, : data collection, data analysis, data interpretation, critical revision

Diane Wintz MD^h^, : data collection, data analysis, data interpretation, critical revision

Kathryn B Schaffer MPH^h^, : data collection, data analysis, data interpretation, critical revision

Dennis J. Zheng MD^i^, : data collection, data analysis, data interpretation, critical revision

Areti Tillou MD^j^, : data collection, data analysis, data interpretation, critical revision

Raul Coimbra MD PhD^j^, : data collection, data analysis, data interpretation, critical revision

Rahul Tuli BS^j^, : data collection, data analysis, data interpretation, critical revision

Jarrett E. Santorelli MD^k^ , : data collection, data analysis, data interpretation, critical revision

Brent Emigh MD^l^, : data collection, data analysis, data interpretation, critical revision

Morgan Schellenberg MD MPH^m^, : data collection, data analysis, data interpretation, critical revision

Kenji Inaba MD^m^, : data collection, data analysis, data interpretation, critical revision

Thomas K. Duncan DO^n^, : data collection, data analysis, data interpretation, critical revision

Graal Diaz PhD^n^ , : data collection, data analysis, data interpretation, critical revision

Erika Tay-Lasso MD^b^, : data collection, data analysis, data interpretation, critical revision

Danielle C. Zezoff MD MBA^b^ , : data collection, data analysis, data interpretation, critical revision

Areg Grigorian MD^b^: literature search, study design, data collection, statistics, data analysis, data interpretation, writing, critical revision
